# Glucocorticoid-Mediated Inhibition of Angiogenic Changes in Human Endothelial Cells Is Not Caused by Reductions in Cell Proliferation or Migration

**DOI:** 10.1371/journal.pone.0014476

**Published:** 2010-12-31

**Authors:** James J. Logie, Sadaf Ali, Kathryn M. Marshall, Margarete M. S. Heck, Brian R. Walker, Patrick W. F. Hadoke

**Affiliations:** Centre for Cardiovascular Science, The Queen's Medical Research Institute, University of Edinburgh, Edinburgh, United Kingdom; Leiden University Medical Center, Netherlands

## Abstract

**Background:**

Glucocorticoid-mediated inhibition of angiogenesis is important in physiology, pathophysiology and therapy. However, the mechanisms through which glucocorticoids inhibit growth of new blood vessels have not been established. This study addresses the hypothesis that physiological levels of glucocorticoids inhibit angiogenesis by directly preventing tube formation by endothelial cells.

**Methodology/Principal Findings:**

Cultured human umbilical vein (HUVEC) and aortic (HAoEC) endothelial cells were used to determine the influence of glucocorticoids on tube-like structure (TLS) formation, and on cellular proliferation (5-bromo-2′-deoxyuridine (BrdU) incorporation), viability (ATP production) and migration (Boyden chambers). Dexamethasone or cortisol (at physiological concentrations) inhibited both basal and prostaglandin F_2α_ (PGF_2α_)-induced and vascular endothelial growth factor (VEGF) stimulated TLS formation in endothelial cells (ECs) cultured on Matrigel, effects which were blocked with the glucocorticoid receptor antagonist RU38486. Glucocorticoids had no effect on EC viability, migration or proliferation. Time-lapse imaging showed that cortisol blocked VEGF-stimulated cytoskeletal reorganisation and initialisation of tube formation. Real time PCR suggested that increased expression of thrombospodin-1 contributed to glucocorticoid-mediated inhibition of TLS formation.

**Conclusions/Significance:**

We conclude that glucocorticoids interact directly with glucocorticoid receptors on vascular ECs to inhibit TLS formation. This action, which was conserved in ECs from two distinct vascular territories, was due to alterations in cell morphology rather than inhibition of EC viability, migration or proliferation and may be mediated in part by induction of thrombospodin-1. These findings provide important insights into the anti-angiogenic action of endogenous glucocorticoids in health and disease.

## Introduction

The well-documented ability of glucocorticoids to inhibit angiogenesis [1] is exploited clinically for the reduction of proliferating capillary haemangiomas [2] and may have potential in treatment of some cancers. For example, exposure of prostate cancer cells to dexamethasone caused a glucocorticoid receptor (GR)-dependent down-regulation of pro-angiogenic factor (vascular endothelial growth factor, VEGF; interleukin-8, IL-8) generation and reduced the size and microvessel density of tumour xenografts [3]. There is also increasing evidence that endogenous glucocorticoids contribute to regulation of new vessel formation (reviewed in [4]). Suppression of angiogenesis may contribute to impaired wound healing in glucocorticoid excess [5] whilst pre-receptor regulation of glucocorticoid concentrations in target tissues (by the isozymes of 11β-hydroxysteroid dehydrogenase; 11β-HSD) has been linked to both physiological and pathophysiological angiogenesis. In the human reproductive tract abnormal inactivation of cortisol by 11β-HSD type 2 may contribute to the dysregulation of angiogenesis associated with heavy menstrual bleeding [6]. Conversely, generation of glucocorticoids by 11β-HSD type 1 has been shown to inhibit recovery from cutaneous wounds and myocardial infarction [7], and increase age-related bone fragility [8] by suppressing angiogenesis.

Surprisingly, despite the extensive use of glucocorticoids as positive controls in many studies of angiogenesis, the mechanisms whereby these steroids inhibit new vessel formation remain unclear. In some cases, inhibition of angiogenesis has been linked to suppression of angiogenic factor generation by cells neighbouring the vasculature [3]; [9]. However, glucocorticoids can directly inhibit tube formation by cultured endothelial cells (EC; [6]). It has not been established whether this is due to the ability of glucocorticoids to inhibit the proliferation, migration and/or remodelling of endothelial cells (which have a central role in angiogenesis [10]). However, since glucocorticoids inhibit proliferation [11]–[13] and migration [14] of vascular smooth muscle cells, a similar effect on endothelial cells seems likely. Indeed, the endothelium is a likely target for glucocorticoids as it expresses both glucocorticoid (GR) and mineralocorticoid receptors [15]; [16]. Furthermore, inhibition of cell protease activity by angiostatic steroids [17] suggests, indirectly, that glucocorticoids inhibit EC migration (since extra-cellular matrix (ECM) degradation is required for cell motility *in vivo*). There is also evidence that glucocorticoids inhibit EC migration in the microvasculature without altering proliferation or viability [18].

Therefore, this investigation built on our previous demonstration [7] that glucocorticoids inhibit angiogenesis *in vitro* (mouse aortic ring in Matrigel), *in vivo* (sub-cutaneous sponge implantation) and during wound healing (following cutaneous incision or myocardial infarction) to explore the hypothesis that this action of glucocorticoids is due to the direct prevention of tube formation by endothelial cells. Complementary *in vitro* techniques were used to determine whether anti-angiogenic effects of glucocorticoids could be attributed to inhibition of endothelial cell viability, migration or proliferation.

## Materials and Methods

### Steroids and drugs

Unless otherwise stated, chemicals, reagents and drugs were obtained from Sigma, Dorset, UK. Enzymes for molecular biology were from Promega, Southampton, UK.

### Endothelial cell culture

Primary human umbilical vein ECs (HUVECs) and human aortic ECs (HAoECs) (Promocell, Heidelberg, Germany) were cultured (37°C, 5% CO_2_) in EC growth medium-2 (EGM-2) consisting of EC basal medium supplemented with 2% fetal bovine serum, gentamicin/amphotericin (GA-1000), and growth supplements (Lonza, Wokingham, UK). All ECs were studied between passages 2 and 6.

### Tube-like structure (TLS) formation

HUVECs and HAoECs (4×10^4^ cells/well) were re-suspended in basal medium (1 ml) and seeded onto 24 well plates coated with Matrigel (250 µl, BD Biosciences, Oxford, UK) as described [19]; [20]. Basal medium comprised EGM-2 supplemented with ascorbic acid, heparin and GA-1000 but without serum or growth factors. Drugs, growth factors or vehicle control (0.004% ethanol v/v) were added at the start of each experiment at concentrations indicated in Figure legends.

Photomicrographs (5× magnification) of the centre of each well were obtained after incubation for 4, 8 and 24 hours [21] and TLSs were quantified by counting branch points by an investigator blinded to treatment. Data were expressed as a percentage of the number of connections in control wells.

TLS formation was assessed directly using time-lapse imaging. HUVECs (2×10^5^) in 2 ml basal medium containing 5 mM HEPES (Lonza, UK) were seeded onto SlideFlasks (Nunc, New York, USA) pre-coated with 750 µl Matrigel and incubated with vehicle or 10 ng/ml VEGF, 600 nM cortisol, or both. Images were acquired with a Leica DM IRBE microscope (10× magnification) and Q500MC image processing system (Leica Cambridge Ltd, UK) from 3 separate positions/flask every 4 minutes for 24 hours. Tubes per field of view were counted every 2 hours in reconstructed movie clips.

### Immunohistological charactisation of TLSs

The endothelial nature of TLSs was assessed using immunohistochemistry. Briefly, HUVECs (10,000) were cultured in standard basal medium (250 µl; 5 hours) on 8-well permanox chamber slides (Nunc, USA) pre-coated with Matrigel (100 µl). TLSs were fixed in 10% formalin and stained using the Vectastain ABC Kit (Vector Laboratories, USA), according to the manufacturer's instructions, in combination with rat anti-mouse CD31 (1 in 50 dilution) monoclonal antibody (BD Pharmingen, USA). Slides were incubated (room temperature; 5 minutes) with 3,3′-diaminobenzidine (DAB) (Vector Laboratories, USA) with positive structures staining brown. For negative controls, the primary antibody was omitted. Slides were viewed by light microscopy (Karl Zeiss Axioskop, Carl Zeiss MicroImaging, Inc, USA) and images captured from a live-feed camera (3-CCD, JVC Professional Europe Ltd, UK) using the Microcomputer Imaging Device (MCID; InterFocus Imaging Ltd, UK).

### Staining of HUVEC cytoskeleton

Components of the EC cytoskeleton under basal conditions and during TLS formation were assessed using immunofluorescent staining. Briefly, HUVECs were re-suspended in EGM-2 basal medium growth medium with or without cortisol (600 nM), and seeded onto coverslips coated with phenol red-free Matrigel (30 µl). After incubation for 1, 4, 10 or 22 hours, cells were fixed, permeabilised and blocked [22], then incubated with mouse monoclonal anti-α-tubulin antibody (clone B512, Sigma; 1∶1000, 1 hour). Secondary goat anti-mouse IgG (H+L, Alexa Fluor 594, Molecular Probes-Invitrogen, Paisley, UK; 1∶500) was then added for 1 hour. Direct fluorescent labelling of filamentous-actin (F-actin) and DNA was achieved by including (1∶500) phalloidin Alexa Fluor 488 (Molecular Probes-Invitrogen) or DAPI (0.1 µg/ml), respectively, with the secondary antibody. Coverslips were mounted on slides with Mowiol-glycerol and imaged as described [22].

### mRNA isolation and identification

ECs or TLSs grown on Matrigel were recovered using MatriSperse cell recovery solution (BD Biosciences, Oxford, UK) according to manufacturer's instructions. RNA was isolated using TRIzol (Invitrogen Life Technologies, Paisley, UK) and quantified with UV spectroscopy. mRNA for GR was assessed in confluent ECs and established TLSs. Total RNA (1 µg) was reverse-transcribed (45 min, 42°C) using a Kit (Promega, Southampton, UK). cDNA templates underwent PCR amplification (35 cycles) with *Taq* polymerase in the presence of oligonucleotides specific for GR (5′-GTGGTTTATAGAGGGCCAAGACTTGG-3′ and 5′-GGCACAACTTCCCTTTTCTGATATACAC-3′). PCR was performed as described [23] with an annealing temperature of 62°C. Products were analyzed by electrophoresis (1.2% agarose gel w/v in TBE buffer containing 0.001% ethidium bromide v/v) and visualized under UV light. PCR product size was 354 base pairs (bp). Negative controls included either no reverse transcriptase, or no cDNA template.

### Quantitative real-time PCR (QrtPCR)

The influence of glucocorticoids on the expression of angiogenic factors during TLS formation was examined using quantitative real-time PCR (QrtPCR). HUVECs (100,000/ml) were seeded on Matrigel-coated cell culture dishes in the presence of cortisol (600 nM) or vehicle (control) and incubated for 1, 4, 8 or 22 hours. RNA was recovered using Matrisperse solution, Trizol (1 ml) was added to the cell pellet and the sample was frozen (−80°C).

cDNA was synthesised from RNA using the Promega Reverse Transcription System (Promega UK, Southampton, UK). The reactions were carried out on an Eppendorf Mastercycler Gradient (Eppendorf, Germany) consisting of incubation at 42°C for 45 minutes followed by 95°C for 5 minutes and finally chilled to 4°C. Quantification of the transcript was performed using human tissue-specific TaqMan primer probe mixes purchased as ready-to-use assays (Applied Biosystems, Cheshire, UK) with the Lightcycler 480 Real Time PCR system (Roche Diagnostics Ltd, West Sussex, UK). Samples were heated to 95°C for 5 minutes for pre-incubation then underwent 50 cycles of PCR amplification (denaturation at 95°C for 10 seconds, primer annealing at 60°C for 30 seconds and elongation at 72°C for 1 second) and finally underwent cooling at 40°C for a further 5 minutes.

RNA levels were determined for each sample, run in triplicate, from standard curves generated for each primer-probe set by serial dilution of pooled cDNA from each tissue. Cyclophylin (Mm02342430_g1; Applied Biosystems, Cheshire, UK) mRNA levels were used as internal references to normalise transcript levels. Changes in mRNA levels in TLS exposed to cortisol were determined for genes involved with stimulation of angiogenesis (VEGF, VEGFR2), inhibition of angiogenesis (thrombospondin-1), cell-matrix interactions (μ_6_-integrin, caveolin-1) and regulation of cell fate (delta-like-4 (Dll4), Notch).

### Endothelial cell viability

ATP production was measured from viable cells (Cell Titer Glo assay, Promega, UK). HUVECs (1.5×10^3^ cells/well) were seeded onto plates for 2 hours, then incubated for 94 hours as described for BrdU. CellTiter-Glo Reagent (100 µl) was then added to each well (10 minutes, room temperature) before detection of luminescence (Wallac 1420 VICTOR^2^ plate reader, Perkin-Elmer, Buckinghamshire, UK).

### Endothelial cell proliferation

To perform BrdU incorporation assays (Calbiochem-Merck, Nottingham, UK), HUVECs (3.5×10^3^ cells/well) were cultured in growth medium (EGM-2 basal medium (Lonza, UK) supplemented with heparin, ascorbic acid, GA-1000, and 2% charcoal-stripped fetal bovine serum) without additional growth factors, and then incubated for 46 hours with: VEGF (25 ng/ml); VEGF plus SU5416 (1 nM-1 µM); VEGF plus cortisol (3 nM-1 µM); vehicle (0.004% ethanol v/v or 0.167% DMSO v/v); or medium alone. BrdU was added to the wells 1 hour after incubations started. After further incubation (45 hours), cells were fixed, denatured (BrdU assay kit, manufacturer's instructions), and incubated with the anti-BrdU antibody and then with horseradish peroxidase-conjugated secondary goat anti-mouse IgG. Finally, cells were incubated with tetra-methylbenzidine (TMB) and the reaction stopped with sulphuric acid (2.5 M). Optical densities (405 nm and 540 nm) were measured (Multiskan Ascent plate reader, Cheshire, UK) and the absorbance of cells without BrdU subtracted from each reading.

### Endothelial cell migration

Boyden chambers [24] were prepared by seeding HUVECs (2×10^5^) onto the upper compartment of 8.0 µm ThinCerts (Greiner Bio-One, Gloucestershire, UK), incubating (24 hours, 37°C), and then labelling with calcein-AM (8 µM). Labelled cells were detached (Trypsin-EDTA) from the underside of ThinCerts and fluorescence measured (Fluoroskan Ascent FL plate reader, Cheshire, UK). The influence of cortisol on migration was assessed by adding VEGF (10 ng/ml), VEGF plus the tyrosine kinase inhibitor SU5416 (1 µM; which inhibits the angiogenic signalling induced by VEGF binding to its receptor), VEGF plus cortisol (600 nM), SU5416 alone or cortisol alone to the medium.

### Statistics

Results from triplicate wells in the same experiment were averaged and treated as single data points. Data are expressed as mean±SEM, where *n* indicates the number of experiments. Statistical analysis was performed by one-way analysis of variance (ANOVA) followed by Dunnett's multiple comparison *post hoc* test or by repeated measures ANOVA. Differences were considered significant when *p*<0.05.

## Results

### TLS formed by HUVECs retain an EC phenotype

**Figure 1:**
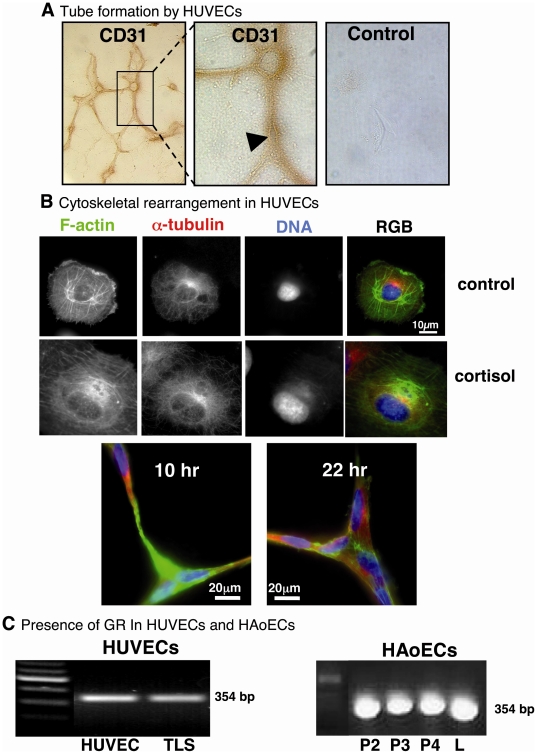
Formation of tube-like structures (TLS) and expression of glucocorticoid receptors (GR) in human endothelial cells. (A) Human umbilical vein endothelial cells (HUVECs) cultured on Matrigel formed a network of tube-like structures (TLSs), after approximately 4 hrs, that retained immunoreactivity for the endothelial cell marker CD31 (original magnification ×10). At higher magnification, cell membranes were evident (arrow head), suggesting development of a lumen in the cell-cell connections (original magnification ×40). No staining was observed in negative controls lacking primary antibody. (B) HUVECs cultured on uncoated cover slips showed clearly-defined cytoskeletal components: filamentous (F)-actin (stained with phalloidin-488; green) and α-tubulin (stained with goat anti-mouse IgG Alexa Fluor 594 secondary antibody; red), and nucleus (DNA stained with 4′,6-diamidino-2-phenylindole dihydrochloride (DAPI); blue). Exposure to cortisol (600 nM; 1 hour) had no apparent effect on microtubule staining but induced a more diffuse and homogeneous distribution of F-actin throughout cell. TLSs stained after 10 hours or 22 hours in culture, consisted of adjoining, filopodia-like extensions, containing both F-actin (green) and α-tubulin (red), connecting neighbouring cells (DNA, blue). (C) GR were detected both in first passage (P1) human umbilical vein (HUVECs) and in passaged human aortic (HAoECs) endothelial cells (P2–P4), by RT-PCR (354 bp product). GR expression was maintained in HUVECs 22 hours after TLS formation. L, Liver (positive control). Negative controls included no reverse transcriptase and no cDNA (not shown).

HUVECs cultured on plastic retained a typical cobblestone appearance (not shown). Once cultured on Matrigel, however, they formed characteristic networks of tube-like structures. These comprised cells connected by filopodia-like extensions which retained immunoreactivity for CD31 (an endothelial cell marker; Figure 1A). At higher magnification these cell-cell connections showed evidence of lumen formation. (Figure 1A).

### Characterisation of cells forming TLSs

Staining of actin filaments (with fluorescently-conjugated phalloidin) and microtubules (with α-tubulin antibodies) clearly identified cytoskeletal fibres in undifferentiated HUVECs (Figure 1B; n = 4). Actin filaments appeared as well-formed, parallel stress fibres throughout the cytoplasm while microtubules appeared to radiate from the centrosome region occupying the traditional peri-nuclear area. F-actin appeared more diffuse and homogeneous throughout cells after 1 hour of cortisol treatment, while microtubule staining appeared unaffected (Figure 1B, n = 2). Microtubules and actin filaments were both observed in TLSs formed after 4 hours on Matrigel (not shown) and remained evident after 8 (not shown), 10 and 22 hours in culture (Figure 1B).

### GR expression in EC monolayers and following formation of TLS by HUVECs

Expression of GR was demonstrated in confluent HUVECs and HAoECs (Figure 1C), and was maintained in HUVECs 22 hours after induction of TLS formation (Figure 1C).

### Cortisol evokes a GR-mediated inhibition of TLS formation

**Figure 2:**
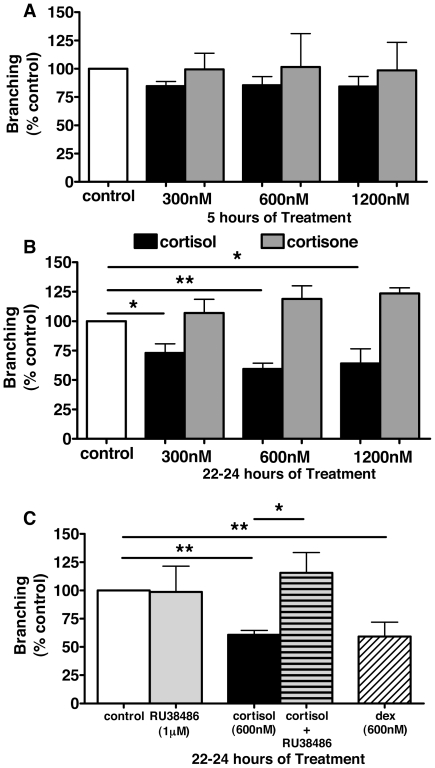
Cortisol, not cortisone, induces glucocorticoid receptor-dependent inhibition of TLS formation by human umbilical endothelial cells. Compared with controls, cortisol, but not cortisone, (300–1200 nM) reduced tube-like structure (TLS) formation by human umbilical vein endothelial cells (HUVECs) after 5 hours in culture (A), an effect which achieved significance after 22–24 hours (B). The glucocorticoid receptor (GR)-selective steroid dexamethasone (600 nM) produced a similar reduction in TLS formation whilst the response to cortisol was abolished by GR antagonism with RU38486 (1 µM) (C). Data represent mean±standard error of mean (SEM) (*n = *3–6, each condition performed in triplicate) and were analysed using one-way analysis of variance (ANOVA) and Dunnett's post hoc test (*p<0.05, **p<0.01).

**Figure 3:**
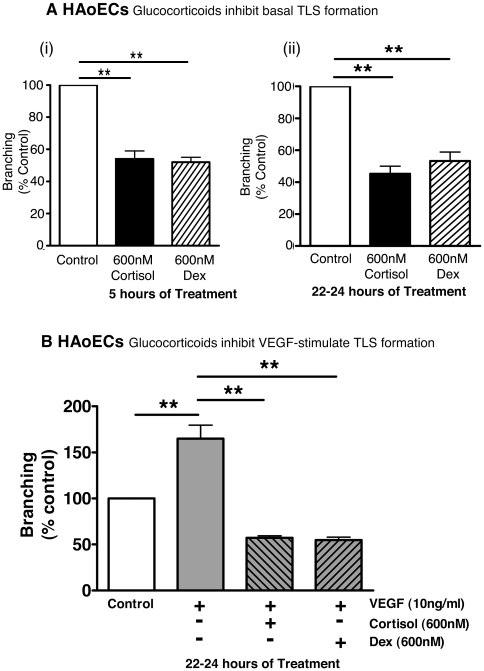
Cortisol and dexamethasone inhibit basal and stimulated TLS formation in human aortic endothelial cells. (A) Cortisol (600 nM) and dexamethasone (600 nM) inhibited basal tube-like structure (TLS) formation of human aortic endothelial cells (HAoEC) after (i) 5 hr and (ii) 22–24 hour treatment. (B) Similarly, growth factor (VEGF)-induced TLS formation by HAoECs was also inhibited after 22–24 hours exposure to cortisol or Dex. Data represent mean±standard error of mean (SEM) (*n = *5, each exposure performed in triplicate) and were analysed by one-way analysis of variance (ANOVA) and Dunnett's post hoc test (*p<0.05, **p<0.01).

TLS formation by HUVECs was inhibited by cortisol (300–1200 nM; Figure 2A–B), but not by cortisone (the inert 11-keto metabolite of cortisol, 300–1200 nM; Figure 2A–B), in a concentration- and time-dependent manner. This inhibition was abolished by the GR antagonist RU38486 (Figure 2C). Inhibition of TLS formation was also obtained with the GR selective, synthetic glucocorticoid dexamethasone (600 nM; Figure 2C). Similar results were obtained in HAoECs in which inhibition of TLS formation by both cortisol and dexamethasone was evident after 5 hours (Figure 3A(i)) and maintained after 22–24 hours (Figure 3A(ii)) in culture.

**Figure 4:**
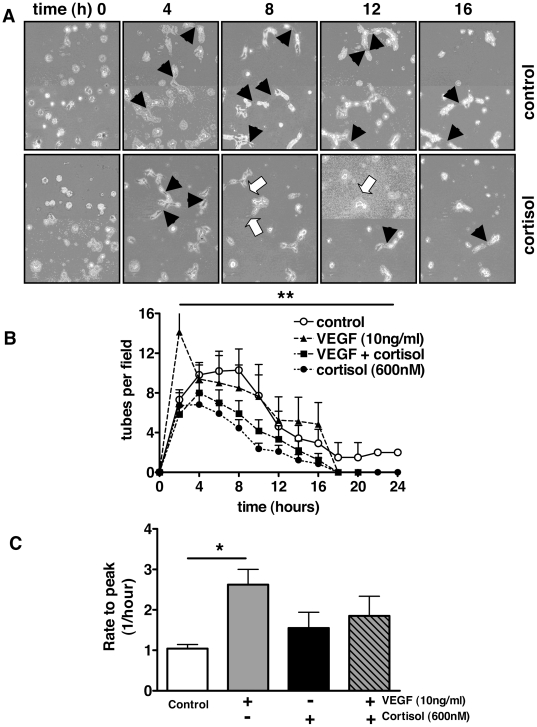
Glucocorticoids reduce formation and stability of TLSs formed by human umbilical vein endothelial cells grown on Matrigel. TLS formation by human umbilical vein endothelial cells (HUVECs) was quantified using time-lapse video microscopy. (A) Tube-like structures (TLS) formation (solid arrows) occurred rapidly (0–4 hours) after seeding, stabilised (4–8 hours) and was followed by degradation and detachment (8–24 hours). (B) Cortisol (600 nM) reduced TLS formation (0–4 hours) and TLS stability (2–4 hours). Exposure to vascular endothelial growth factor (VEGF, 10 ng/ml) increased and accelerated TLS formation but did not influence TLS stability. This effect of VEGF was abolished by co-incubation with cortisol (600 nM) (**p<0.01 by repeated measures analysis of variance (ANOVA)). (C) The rate to maximum TLS development was increased by VEGF (*p<0.05 by one-way ANOVA and Dunnett's post hoc test) and this effect was blocked by cortisol. Data represent mean±standard error of mean (SEM) (*n = *6).

VEGF stimulated TLS formation both by HAoECs (Figure 3B) and by HUVECs (Figure 4). This effect was blocked in both cell types by cortisol and dexamethasone (*p*<0.01) after 4 hours (data not shown) and 22–24 hours (Figures 3 & 4) in culture. PGF_2α_ (100 nM) also stimulated TLS formation by HUVECs (138±9% connections relative to control, *p*<0.01) and this effect was blocked by 600 nM cortisol (105±8% connections, *p*<0.05 compared with PGF_2α_ alone) after 5 hours.

Time-lapse imaging of HUVECs cultured on Matrigel revealed minimal cell migration or proliferation, with TLS formation consisting mainly of cell stretching to facilitate connection between adjacent cells. TLS generation began almost immediately after seeding cells on Matrigel, and peaked after approximately 8 hours (Figure 4A–4B), followed by degradation and detachment. Most cells had detached after 24 hours. Cortisol (600 nM) reduced TLS formation (Figure 4B), whereas VEGF (10 ng/ml) stimulated TLS formation and this effect was blocked by cortisol (600 nM). These observations were confirmed by comparison of the time taken to achieve maximum tube formation (Figure 4C).

### Influence of cortisol on endothelial cell migration, proliferation and viability

**Figure 5:**
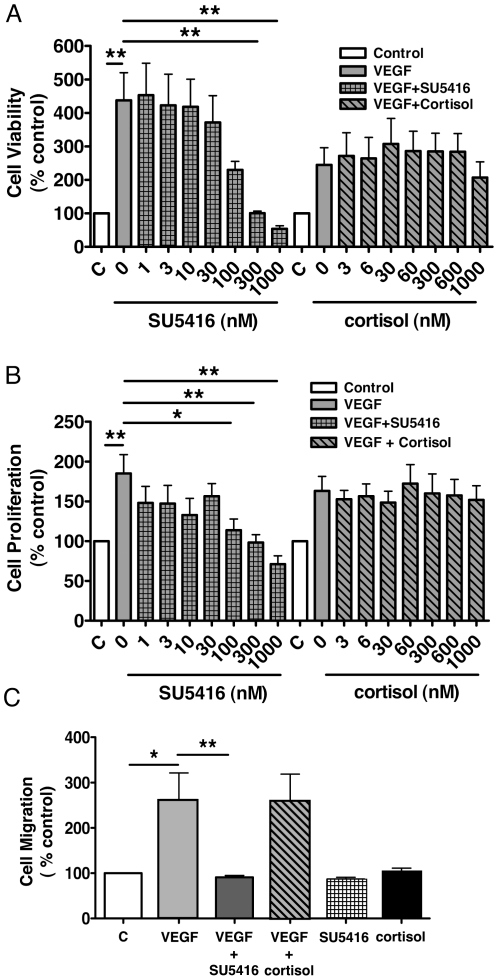
Cortisol does not inhibit human umbilical vein endothelial cell migration, proliferation or viability. (A) Human umbilical vein endothelial cell (HUVEC) viability was increased by VEGF (25 ng/ml) and this effect was blocked in a concentration-dependent manner by (1–1000 nM) SU51416 but not by (3–1000 nM) cortisol (B) Similarly, VEGF (25 ng/ml)-stimulated proliferation of cultured HUVECs was blocked in a concentration-dependent manner by (1–1000 nM) SU51416 but not by (3–1000 nM) cortisol. (C) Vascular endothelial growth factor (VEGF) (10 ng/ml; 24 hr 37°C)-stimulated migration of (HUVECs) was abolished by (1 µM) SU51416 but not by (600 nM) cortisol. Data represent mean±standard error of mean (SEM) (*n* = 6, each condition performed in triplicate); *p<0.05 and **p<0.01 analysed by one-way analysis of variance (ANOVA) and Dunnett's post hoc test.

The impact of exposure to cortisol on cell viability was tested by measuring ATP production. Exposure to VEGF increased ATP production by HUVECs. ATP production was inhibited in a concentration-dependent manner by SU5416 (1 nM-1 µM) but not by cortisol (3 nM–1 µM) (Figure 5A). Cell proliferation was measured using BrdU incorporation. Exposure to cortisol (3 nM-1 µM) did not alter the VEGF-induced increase in BrdU incorporation whereas SU5416 (1 nM–1 µM) produced a concentration-dependent inhibition (Figure 5B). Migration of HUVECs was assessed using a modified Boyden chamber assay. The EC_50_ (10 ng/ml) for VEGF-stimulated HUVEC migration was established in pilot investigations (not shown). VEGF-induced migration was abolished by exposure to SU5416 whereas cortisol (600 nM) had no effect on either basal or VEGF-induced migration (Figure 5C).

### Influence of cortisol on gene expression of potential modulators of angiogenesis in HUVECs

**Figure 6:**
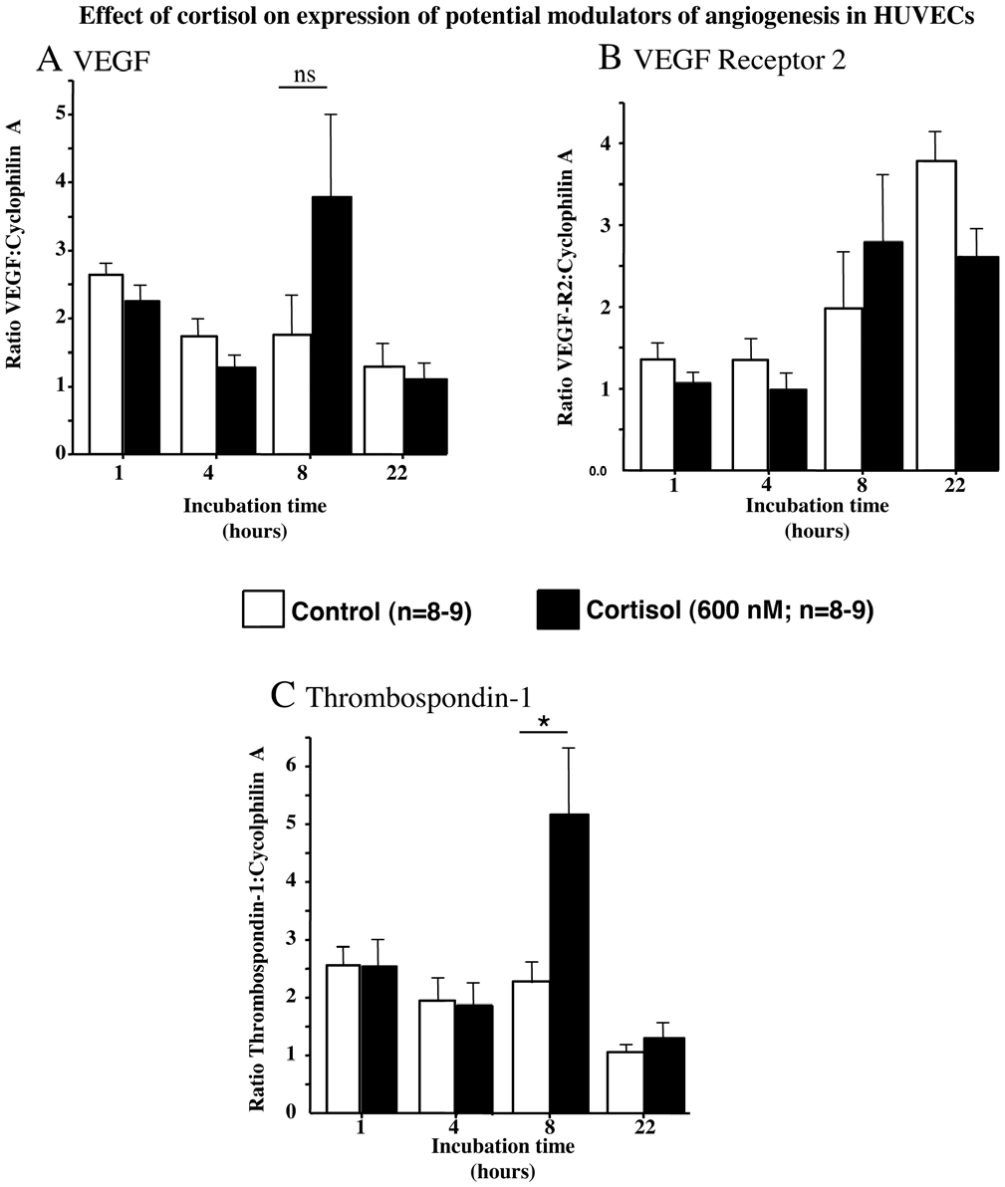
Cortisol increases expression of thrombospondin 1, but not of VEGF or VEGF receptor 2 during tube-like structure formation by HUVECs. Effects of cortisol (600 nM) on the expression of (A) vascular endothelial growth factor (VEGF), (B) vascular endothelial growth factor receptor 2 (VEGFR2) and (C) thrombospondin-1 (TSP-1) during tube-like structure (TLS) development by human umbilical vein endothelial cells (HUVECs). Cortisol (600 nM) added to the culture medium at time of cell seeding, did not alter expression of VEGF or VEGFR2 but transiently increased (*p<0.05) TSP-1 expression after 8 hours in culture. Quantitative real-time PCR (QrtPCR) measurements; were expressed as a ratio of concentration of mRNA of gene of interest to internal control (cyclophilin A; *n = *8–9, with each condition performed in triplicate).

QrtPCR measurements indicated gene expression varied with time during TLS development but that cortisol did not alter mRNA levels of VEGF (Figure 6A) or VEGF-R2 (Figure 6B). Similarly cortisol had no effect on Dll-4, notch-1, α_6_-integrin or caveolin-1 (not shown). In contrast cortisol produced a significant (*p*<0.05) 2.3-fold increase in thromobospondin-1 mRNA level in TLSs after (but not 1, 4 or 22) 8 hours incubation (Figure 6C).

## Discussion

This investigation addressed the hypothesis that the potent anti-angiogenic action of glucocorticoids is due to prevention of tube formation by endothelial cells. These results show that glucocorticoids do indeed induce direct, GR-mediated inhibition of tube formation by primary human ECs. Application of time-lapse imaging clearly demonstrated that TLS formation by ECs cultured on Matrigel required extension of filopodia and formation of connections with minimal EC proliferation or migration. Exposure to glucocorticoids reduced the formation of cell-cell contacts rather than increasing degradation of existing tubes. Consistent with this mode of action, glucocorticoid exposure did not measurably impair EC proliferation, migration or viability. Furthermore, glucocorticoids inhibited basal, and VEGF- or PGF_2α_-stimulated, angiogenesis, suggesting that they act by inhibiting the final common pathway which initiates tube formation. The majority of experiments used human umbilical vein endothelial cells, which provide a useful and readily available source of cells. However, key results were recapitulated using human aortic endothelial cells, demonstrating that the actions of glucocorticoids were not restricted to a single type of endothelial cell, but are likely to be relevant to ECs located throughout the systemic circulation.

Previous investigations suggest that glucocorticoids inhibit angiogenesis by inhibiting VEGF [25]–[28] and/or prostaglandin [29]–[31] production. In the present study we found that glucocorticoids blocked basal, VEGF-induced, and PGF_2α_-induced, TLS formation. It may be that basal TLS formation in this system is stimulated by release of VEGF and prostanoids from the Matrigel and/or the ECs. In this way glucocorticoids could be interfering with either VEGF or prostanoids under basal conditions, as well as after addition of growth factors. Since VEGF and PGF_2α_ stimulate angiogenesis through discrete second messenger pathways (phospholipase Cγ-Ras-Raf [32] and cyclic adenosine monophosphate (cAMP)-inositol trisphosphate [33], respectively), it appears likely that glucocorticoids influence a final common pathway (*e.g.* extracellular signal-regulated kinase-mitogen activated protein kinase (ERK-MAPK)) involving calcium exchange and/or its downstream signalling effects. Therefore, we explored ‘downstream’ effects on cell morphology, migration and proliferation.

Few previous studies have investigated the effects of glucocorticoids on EC biology *in vitro*. Those that are available have generally used pharmacological concentrations of synthetic glucocorticoids [34] or ECs of non-human origin [35]. The receptor responsible for angiostatic effects of corticosteroids has not been well characterised. GR and mineralocorticoid receptors have both been demonstrated previously in HUVECs [36]; [37], but neither receptor has been identified in HAoECs. We used a qualitative approach to show that the GR receptor is present in HUVECs and in HAoECs. The ability of the GR-selective steroid dexamethasone to inhibit TLS formation, combined with the attenuation of cortisol-mediated inhibition by a GR antagonist (RU38486), indicates that glucocorticoids directly inhibit TLS formation by activation of GR.

Using time-lapse imaging to examine the dynamic nature of TLS formation indicated that, consistent with other endothelial tube formation models [38]; [39], cell proliferation and migration play little role in TLS development *in vitro.* HUVEC viability was not reduced by increasing concentrations of cortisol, indicating that reduced TLS generation is not the result of a toxic effect on ECs. Moreover, we demonstrated that glucocorticoids do not measurably inhibit migration or proliferation of HUVECs. The ability of glucocorticoids to inhibit EC migration has not been assessed before but has been inferred from the inhibitory action of angiostatic steroids on protease activity [40]. Glucocorticoid-mediated inhibition of proliferation has been demonstrated in rat and bovine smooth muscle cells [11]–[13] but previously reported effects on ECs are inconsistent: high concentrations of synthetic corticosteroid inhibit human EC proliferation *in vitro* [41]–[43] but dexamethasone (10^−10^–10^−5^ M) had no effect on bovine corneal EC proliferation [44]. The lack of effect of cortisol in the proliferation assay (Figure 5B) is supported by data generated by time-lapse imaging with direct observation of endothelial cell proliferation which showed that proliferation is not a key component of TLS formation in this model.

Time-lapse imaging also revealed that formation of TLSs occurred via the development and connection of filopodia-like extensions between adjacent cells and that cortisol inhibits initial formation of tubes, rather than accelerating their degradation. Imaging of the cytoskeleton in HUVECs supported this hypothesis, demonstrating that the arrangement of F-actin, but not α-tubulin, became rapidly disorganised in cells treated with cortisol. Dexamethasone-mediated alterations in the cytoskeleton have been reported previously in EC monolayers (40) and it is known that interfering with microfilaments or microtubules suppresses key angiogenic responses (41). These results suggest that glucocorticoids may alter EC morphology or the ability of endothelial cells to interact with one another through the establishment of productive cell-to-cell connections. However, real time analysis of suggests that this is not the only mechanism through which cortisol inhibits tube formation.

The lack of effect of cortisol on Dll-4, notch, caveolin-1 and α_6_-integrin suggests that increased expression of these factors does not contribute to the GR-mediated inhibition of TLS formation. Furthermore, although glucocorticoids decrease stimulated expression of VEGF (via GR) in some cells (including vascular smooth muscle (24)), the current results suggest that (in the absence of additional growth factors) reduced VEGF expression in endothelial cells does not contribute to cortisol-mediated inhibition of TLS formation. This is supported by the lack of effect of cortisol on VEGF-R2 mRNA levels, which is consistent with a previous study in cell monolayers [45]).

Indeed, of the factors examined, only TSP-1 mRNA was shown to be regulated by cortisol. The magnitude of this induction was similar to that produced by glucocorticoids, in trabecular meshwork (TM) cells (which have similarities to vascular endothelial cells; (43)). Since it is a recognised inhibitor of tube formation (44, 45), it is conceivable that increased expression of TSP-1 could contribute to the anti-angiogenic properties of glucocorticoids. Certainly, GR-dependent regulation of TSP-1 mRNA synthesis and protein production has been reported (44). In addition, our group has reported evidence that TSP-1 contributes to cortisol-mediated inhibition of angiogensis in human uterine endothelial cells (6).

In summary, although the phenomenon of steroid-induced inhibition of angiogenesis has been recognised for over 30 years, its mechanism(s) of action remain(s) obscure [46]. The present study indicates a direct, GR-dependent action on ECs, independent of the anti-inflammatory effects of glucocorticoids. Cortisol-mediated inhibition of tube formation was shown to occur without a measurable reduction in endothelial cell migration or proliferation. Investigations of into the mechanism of GR-mediated inhibition of TLS formation suggest that alterations in cytoskeletal structure and induction of anti-angiogenic TSP-1 contribute to this process. Our results shed light on the mechanism(s) underlying glucocorticoid-mediated inhibition of angiogenesis in subcutaneous sponge implants *in vivo*, and in wound healing following skin incision or myocardial infarction [7], and strengthen the case for pharmacological inhibition of glucocorticoid action in these physiological conditions. Our previous demonstration of increased angiogenesis in animals with genetic disruption of (the glucocorticoid-generating enzyme) 11β-HSD1 (7) suggest that these *in vitro* data obtained with concentrations of cortisol approaching high physiological levels are relevant *in vivo*.
